# Impact of Plant Cover on Fitness and Behavioural Traits of Captive Red-Eyed Tree Frogs (*Agalychnis callidryas*)

**DOI:** 10.1371/journal.pone.0095207

**Published:** 2014-04-16

**Authors:** Christopher J. Michaels, Rachael E. Antwis, Richard F. Preziosi

**Affiliations:** Faculty of Life Sciences, University of Manchester, Manchester, United Kingdom; Smithsonian's National Zoological Park, United States of America

## Abstract

Despite the importance of *ex situ* conservation programmes as highlighted in the Amphibian Conservation Action Plan, there are few empirical studies that examine the influence of captive conditions on the fitness of amphibians, even for basic components of enclosure design such as cover provision. Maintaining the fitness of captive amphibian populations is essential to the success of *ex situ* conservation projects. Here we examined the impact of plant cover on measures of fitness and behaviour in captive red-eyed tree frogs (*Agalychnis callidryas*). We found significant effects of plant provision on body size, growth rates and cutaneous bacterial communities that together demonstrate a compelling fitness benefit from cover provision. We also demonstrate a strong behavioural preference for planted rather than non-planted areas. We also assessed the impact of plant provision on the abiotic environment in the enclosure as a potential driver of these behavioural and fitness effects. Together this data provides valuable information regarding enclosure design for a non-model amphibian species and has implications for amphibian populations maintained in captivity for conservation breeding programmes and research.

## Introduction

Amphibian species are globally threatened as a result of synergistic threats including disease, over-collection, habitat loss and degradation, and climate change [1], [2]. *Ex situ* conservation has been identified as a major component of the conservation response, and the only possibility for some species that are declining rapidly in the wild [3], [4]. Successful *ex situ* conservation and reintroduction programmes comprise population ‘rescue’ from the wild, breeding of healthy offspring, and (re)establishment of self-sustaining wild populations once threats have been alleviated [5]. Long-term success of captive breeding programmes depends on the ability to maintain populations that can be sustained over many generations and that persist when reintroduced to the wild. However, very little empirical evidence is available about the effect of captive conditions, such as diet and environment, on fitness traits in amphibians.

Environmental complexity, and specifically shelter or cover provision, may be an important component of enclosure design and captive husbandry for amphibians. Wild amphibians use cover for a variety of reasons, including behavioural homeostasis [6], ambush feeding [7] and predator avoidance [8]-[10]. Amphibians in captivity are likely to benefit from cover provision as it provides environmental complexity, abiotic gradients that allow behavioural homeostasis, and refugia that allow captive amphibians to shelter from perceived predation risk as a result of natural ‘hardwired’ behaviours [7].

Existing research on effects of captive environmental conditions, although limited, is biased towards laboratory populations of *Xenopus laevis* [11]-[16], with only one [7] out of a total of six studies using amphibians that are not laboratory model species. In *Xenopus*, shelter seems to confer behavioural benefits, but has no impact on growth or body condition [11]–[16], while in non-model taxa it may have positive effects on growth as well as behaviour [7].

Current research in amphibian husbandry tends to use measures of growth rates, body condition and behaviour to assess fitness effects. Captive husbandry protocols may also have more subtle effects on amphibian health and fitness. Bacterial communities living on the skin of amphibians are emerging as an important component of amphibian immunity (reviewed in [17]). The skin of amphibians provides symbiotic bacteria with nutrients and an environment in which to reproduce, and in return these can protect the host from pathogenic infection by competing for space and resources, altering the microenvironment on the amphibian skin to prevent colonisation by pathogens, and through the production of anti-microbials that kill or inhibit the growth of pathogens [18]–[20]. Given the current lack of knowledge about amphibian resistance to emerging diseases, it may be important to consider the diversity and stability of the symbiotic skin bacterial community when reintroducing captive amphibians to the wild. Amphibians gain bacteria through interaction with conspecifics and via transmission from the environment – both of which are controlled by husbandry protocols in captivity [18], [21]–[23]. Captive husbandry may also affect symbiotic bacteria by altering the abiotic environment to which the amphibian and the bacteria are exposed, including temperature, humidity and ultraviolet radiation levels. It has previously been shown that captive diet (carotenoid-enriched and carotenoid-free diets) has a significant effect on the bacterial communities of red-eyed tree frogs (*Agalychnis callidryas*), and therefore other facets of captive husbandry may do so too [24].

Here we investigate the effect of plant cover on growth rates, body condition, and symbiotic bacterial communities of captive *Agalychnis callidryas*. We also use behavioural assays to assess environmental preference, and characterise the effects of shelter on the environmental parameters in frog enclosures.

## Methods

### Ethics statement

All methods used in this study were non-invasive and did not require a UK Home Office Licence. The University of Manchester Ethics Committee approved this study prior to commencement.

### Study species

We used *Agalychnis callidryas* in this study. This species is widely held in both public and private collections, as well as being closely related both phylogenetically and ecologically to threated phyllomedusine frogs including *A. moreletii*, *A. annae and A. lemur*. Being an arboreal frog, it is dependent on enclosure furnishings for shelter in captivity and cannot shelter in the substrate as do most terrestrial anurans. Moreover, this species has been the focus of several investigations into other aspects of captive husbandry [24], [25] and the effects of size and body condition on fitness components in the wild are relatively well understood [26], [27].

### Group 1 frogs: environmental parameters, growth and body condition and bacterial communities

#### Study animals and husbandry

Group 1 consisted of frogs (N = 19) reared from metamorphosis in planted or non-planted enclosures. These frogs were used to examine environmental variables, growth and body condition and bacterial communities. Frogs were raised from a single clutch of eggs produced by a pair of captive adult frogs. Tadpoles were reared in a single large aquarium (100×50×30 cm) at 23–25°C. Tadpoles were provided with plastic plants for shelter and water quality was maintained using an air-driven sponge filter and partial water changes. Tadpoles were fed a diet of crushed fish flakes (Tetramin) and dried powdered *Spirulina* algae in a 1∶1 ratio. After metamorphosis, froglets were randomly assigned to experimental enclosures (before day 45: ExoTerra ‘Faunarium Medium’; after day 45: ExoTerra ‘Reptarium Mini/Tall’). Enclosures were housed in a climate-controlled room at the University of Manchester at a diurnal ambient temperature of 24°C and nocturnal ambient temperature of 22°C, and an ambient relative humidity of 60–80%. Circulation fans prevented air stagnation. Reptisun 10.0 UVB fluorescent tubes (ZooMed Inc.) and plant growth tubes (Alto Universal T8 fluorescent tubes, Philips) with reflectors (Arcadia) were provided on top of terraria on a 12∶12 hr light cycle. All UVB bulbs were subject to a 100 hour burning-in period before use to reduce emission fluctuations associated with new tubes, and replaced within one year of use (F. Baines, pers. comm.).

All enclosures contained a small water dish and a substrate of moistened paper towels, frequently used as a substrate for *Agalychnis* frogs [28]. Enclosures were sprayed once daily, and water dishes were cleaned and refilled when soiled (or at least every other day). Substrate was changed and vivaria wiped down weekly or more frequently if soiled. Gloves were worn during cleaning to avoid introduction of any novel bacteria. Any necessary disinfection of enclosures was undertaken using F10 (Health and Hygiene) diluted 1∶500 with water. Frogs were fed exclusively on black crickets (*Gryllus bimaculatus*), following Ogilvy et al. [25], and gut-loaded using a rotation of fresh fruit and vegetables, dry instant porridge (ReddyBrek) and commercial ‘bug-grub’ (Nutrogrub, Vetark). Appropriate cricket size was judged as approximately the distance between the eyes of frogs. Food was offered daily for small juveniles, decreasing to 3 times weekly in adults. Crickets were dusted with Nutrobal powdered dietary supplement (Vetark) before every feeding for juveniles and sub-adults and every other feeding for adults. Crickets were added to enclosures as close to lights-out as possible to maximise powder retention until crickets were eaten. Food was provided in quantities that ensured a small number of uneaten crickets remained in enclosures the morning after feeding, indicating frogs were fed to satiation.

#### Experimental design

Enclosures were either planted (n  =  9 frogs) or non-planted (n  =  10 frogs), with three enclosures per treatment group. Sample sizes were not equal as one froglet died at metamorphosis. Froglets were allocated alternately to treatment enclosures on metamorphosis and enclosure types were positioned alternately on the same shelf. Planted enclosures contained living plants (*Sciandapsus sp*. – ‘Devil’s ivy’), with no other cover provided in either enclosure type. On day 180 (after the final growth and body condition measurements were taken), *Dieffenbachia* plants were introduced into all tanks, and *Sciandapsus* plants removed (see ‘bacterial community data’ below) as the larger leaves provided a better resting site for sub-adult frogs.

### Environmental data

#### Thermal imaging of enclosures

A FLIR 5 Infrared camera (FLIR Systems) was used to thermally image planted (*Sciandapsus sp*.) and non-planted enclosures (ExoTerra ‘Faunarium Medium’).

### Skin temperatures of frogs and position preference

A FLIR 5 Infrared Camera (FLIR Systems) was used to measure the skin temperature of frogs (N = 19) under planted and non-planted conditions. Measurements were taken on three consecutive days at 14:00, when terraria had reached their peak daytime temperature. Mean values were calculated for each frog over the three days and t-tests were used to compare mean skin temperatures. At the same time, tanks were visually split into thirds (top, middle or bottom) and the height of the frogs’ resting positions was recorded as top, middle or bottom (recorded as values 1, 2, and 3 respectively).

#### Evaporation rates

Four extra of each planted and non-planted enclosures (ExoTerra ‘Faunarium Medium’) were set up identically to those used in this experiment with the exception that frogs were not housed in them. The drying rate of the enclosure was assessed by saturating paper towel substrate in enclosures maintained at 25°C with 100 ml of water and measuring weight loss of the entire paper towel to the nearest gram after 2, 4, 6, 8 and 24 hours. Rate of water loss was calculated for each enclosure and the data was analysed using a Mann-Whitney U-test.

#### UV index

Planted and non-planted vivaria (ExoTerra ‘Reptarium Mini/Tall’) housing Group 1 experimental frogs were used for the measurement of UV index (UVi). UVi is a unitless measure of UVB radiation, weighted for its biological significance, and is becoming widely used in the study of UVB exposure in herptiles (e.g. [29]). The Reptisun 10.0 UVB fluorescent tube was located across the back portion of the top of the tank. Measurements were taken from all three planted and all three non-planted enclosures using a Solarmeter 6.5 (Solartech Inc.) at three heights (top – directly beneath the mesh; middle – half-way down the enclosure; and bottom – as close to the substrate as the measuring device would allow) and at four positions at each height (back left, back right, front left, front right). Measurements were taken on only one day, as conditions did not vary across time. Readings from the front top were discarded as uninformative (they showed negligible readings not consistent with the height in the tank due to the fluorescent tubes being positioned across the back top of enclosures). A GLM with the model UVi  =  Treatment + Height + Treatment*Height was used for analysis (height was treated as categorical; top, middle, bottom).

### Growth and body condition data

Frogs were photographed three times each against a scale after 6 (once tail had fully disappeared), 90 and 180 days post metamorphosis. Image J (freeware available at http://imagej.nih.gov/ij/) was used to calculate the snout-to-vent length (SVL) of frogs, and the mean taken from the three measurements of each individual. The mass of each frog was recorded on days 90 and 180, subsequent to a four-day fasting period (the routine maximum period between feedings in the husbandry schedule) to avoid variation in mass due to foraging success [30], [31]. Gloves were worn during handling to avoid damage to frog skin or the introduction of novel bacteria. A body condition index (BCI) was calculated for each individual at days 90 and 180 using the formula; BCI  =  M [L_0_/L]^R^ where M is the mass, L is the snout-vent length for a given individual, L_0_ is the arithmetic mean of the SVL's for the whole population, and R (the scaling component) is equivalent to the regression value (R value) of M on L for the whole population [32], [33]. This measure of body condition allows for the allometric relationship between length and mass [32] and has been found to accurately represent actual body condition and energy reserves in amphibians [33]. GLMs were used to control for effects of enclosures in order to validate treatment of individuals as experimental units (see Results).

### Bacterial community data

Frogs were swabbed twice for cutaneous bacterial communities. The first swabs were taken while frogs were being maintained under planted (*Scindapsus sp.*) or non-planted conditions; henceforth ‘Phase 1’ swabs. The second set of swabs were taken two months after *Dieffenbachia* plants were added to all enclosures, after the end of the planted/non-planted study; henceforth ‘Phase 2’ swabs.

The dorsal and ventral regions of the body were sampled separately to maximise coverage. Sterile powder-free nitrile gloves were worn during swabbing and changed between frogs to minimise cross-contamination. Frogs were rinsed twice on their dorsal surface using sterile bottled water to remove any transient (i.e. non-symbiotic) bacteria from their skin [34], and then swabbed all over their dorsal surface to collect cutaneous bacterial communities using sterile Eurotubo collection swabs (Deltalab, Rubi, Spain). Swabs were placed into 1.5 ml sterile Eppendorf tubes containing 1 ml of 0.8% w/v (i.e. 1 M) NaCl_2_ solution. The rinsing and swabbing process was repeated for the ventral surface of each frog. Care was taken to ensure frogs were not harmed during this process, and individuals monitored for two weeks post-swabbing for signs of distress or injury in response to the swabbing, of which none were detected.

Eppendorf tubes containing swabs were vortexed to dissociate bacteria from the swab. The swab was removed and samples were serially diluted by pipetting 100 µl into 900 µl of 0.8% w/v NaCl_2_ solution up to a concentration of 10^-2^ for each sample. 100 µl of each dilution (10^−1^ and 10^−2^) were plated out on R2A agar media (Lab M Ltd., United Kingdom) and incubated at 25°C (the same temperature at which frogs were maintained). New morphologically distinct bacteria colonies (‘morphotypes’) were counted up until 12 days post swabbing, after which negligible new colony growth was observed.

Bacterial counts were multiplied by their respective dilution factors and averaged for each morphotype. Data for the dorsum and ventrum of each frog were combined, and differences in the overall bacterial community composition during each phase were analysed using the Adonis function in the Vegan package in RStudio. Adonis is a permutational multivariate analysis that uses distance matrices to analyse the variation in the overall bacterial community structure associated with frogs according to treatment group. The effect of treatment group on species richness (the number of different morphotypes on each individual) and total abundance (total number of cultured bacteria for each individual – used as a proxy for bacterial load of frogs) was analysed using t tests in JMP 10.

Representative colonies of each morphotype were streaked out on R2A agar until a pure culture was obtained. Bacterial species were identified using 16S rDNA sequencing with universal primers 27F (5′-GTGCTGCAGAGAGTTTGATCCTGGCTCAG-3′) and 1492R (5′-CACGGATCCTACGGGTACCTTGTTACGACT-3′) [35]. 16S rDNA fragments were obtained through colony PCR amplification using the Platinum PCR SuperMix (Invitrogen, Life Technologies) according to the manufacturer's instructions. DNA fragments were amplified by PCR using the following programme: 95 °C for 2 minutes followed by 35 cycles of 94 °C for 30 s, 55 °C for 30 s, and 72 °C for 90 s, with a final extension step of 5 minutes at 72 °C. Prior to purification and sequencing PCR products were checked for the correct length with gel electrophoresis. PCR products were purified with the GenElute PCR Clean-up Kit (Sigma-Aldrich). PCR products were sequenced at the DNA Sequencing Facility, University of Manchester, UK. A consensus sequence was obtained by combining the forward and reverse sequences in DNA Dynamo Sequence Analysis Software (BlueTractorSoftware Ltd., UK). Consensus sequences were then blasted against the NCBI database (http://blast.ncbi.nlm.nih.gov/Blast.cgi) to identify each morphotype to genus level. Morphotypes with 99% sequence similarity or greater were considered the same species [36].

### Group 2 frogs: behavioural data

Adult frogs previously reared under the same conditions as one another were maintained in ‘home’ enclosures for 2 weeks with (n = 15) or without plants (*Scindapsus*; n = 15) as their ‘prior treatment’. Frogs were then transferred individually to anexperimental arena for four days, starting on day 185 after metamorphosis. The arena consisted of a glass terrarium (90×45×45 cm) with one end furnished with artificial terrarium plants and the other no plants. A line was drawn at the centre of the arena and the water bowl positioned to exactly straddle the line on the floor. Substrate was damp paper towels. Frogs were allowed to acclimatise for one night and then for the following three days the position of the frog in the tank was scored twice a day (10:00 and 16:00 hrs). Frogs were scored ‘1’ if they were positioned at the end of the arena with live plants, and ‘0’ if they were at the end with artificial plants. A frog exactly straddling the centre of the tank would have been scored 0.5, although this never occurred. Mean scores over the three days were calculated and analysed using a non-parametric Wilcoxon signed rank test.

## Results

### Environmental data

**Figure 1:**
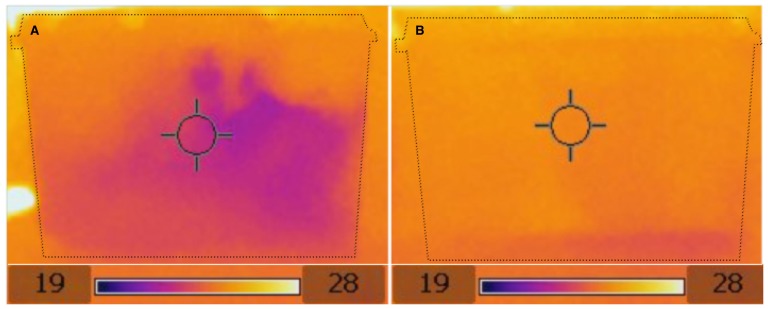
Thermal imaging pictures of planted (A) and non-planted (B) enclosures, showing the greater thermal heterogeneity of planted enclosures. Dotted lines indicate the limits of enclosures. Scale is in °C.

The presence of plants in the enclosure creates a wider variety of thermal microclimates in the vivarium (Figure 1A and 1B). In non-planted enclosures temperatures are uniform at around 26°C except for the paper towel substrate, which is cooler around the water dish at approximately 24°C (Figure 1B). Conversely, a range of warm and cool spots exist throughout the planted enclosures, with temperatures ranging from approximately 22–26°C (Figure 1A). Planted enclosures were also found to dry significantly slower than non-planted enclosures (Table 1).

**Table 1:** Statistical comparisons of environmental parameters in planted and non-planted frog enclosures.

Variable	Planted Value	Non-Planted Value	Test used	Test statistic	d.f.	P
Mean skin temperature	26.76°C	26.33°C	2-tailed T	t = 0.863	18	0.411
Variance in skin temperature	2.050	0.173	Hartley's F_max_	F_max._ = 11.867	8	<0.05
Mean height position score	2	3	2-tailed T	T = 3.00	18	0.017
Number of frogs in top, middle and bottom of enclosures	Top: 4 Middle: 1 Bottom: 4	Top: 10 Middle: 0 Bottom: 0	G test of likelihood ratio	χ^2^ = 9.535	2	0.0085
Evaporation rate	1.02 ghr^−1^	1.25 ghr^−1^	Mann-Whitney U	U = 0.00	7	0.029

**Figure 2:**
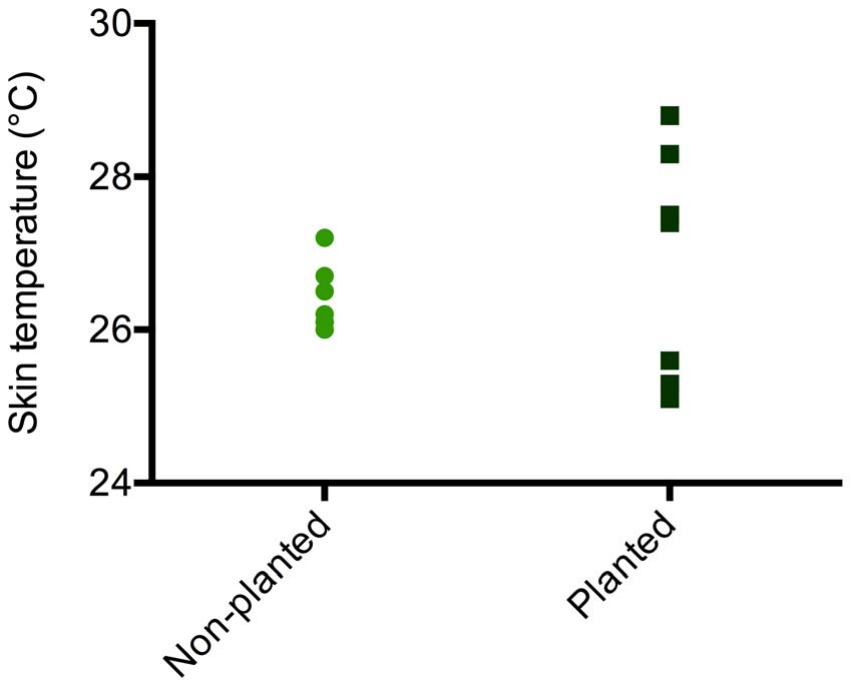
Skin temperatures of frogs housed under planted and non-planted conditions. Mean skin temperature is not significantly different between treatments (p  =  0.411), but frogs in planted enclosures show a significantly greater variance in skin temperature (p < 0.05).

Frogs in planted and non-planted environments did not have significantly different mean skin temperatures (Table 1, Figure 2). However, frogs sat at more varied resting heights in planted environments and consequently displayed a significantly larger variance in skin temperature than animals in non-planted enclosures (Table 1, Figure 2).

UVi was significantly influenced by position (top, middle, bottom) in the tank (F_1,4_  =  839.983, p < 0.001), with UVi falling in a steep gradient with increasing distance from the lamp. UVi was also predicted by treatment (F_1,4_  =  15.205, p < 0.001), with planted tanks having a steeper UVi gradient. Mean UVi was not significantly different between treatments immediately under the lamp (t_5_  =  2.500, p  =  0.808), but became significantly lower in planted setups at the middle and bottom of the tank (middle: t_5_  =  2.404, p  =  0.027; bottom: t_5_  =  6.484, p < 0.001).

### Growth and body condition

**Figure 3:**
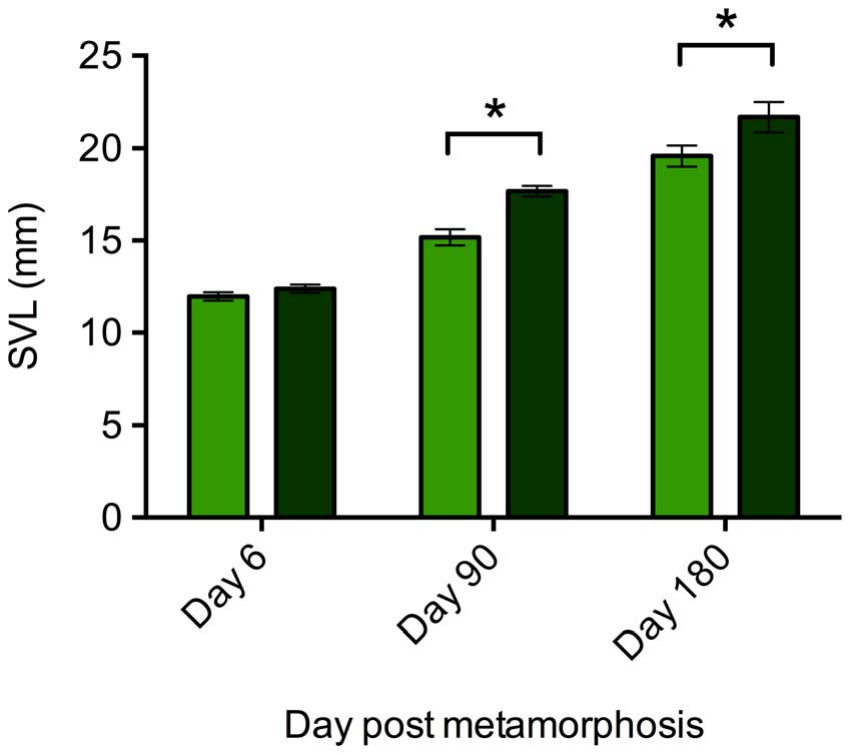
SVL of frogs under planted (dark green) and non-planted (light green) conditions at the beginning of the study, and at 3 months and 6 months after. Error bars show ±1 S.E.M. An * indicates a significant (p<0.05) difference between treatment groups.

**Figure 4:**
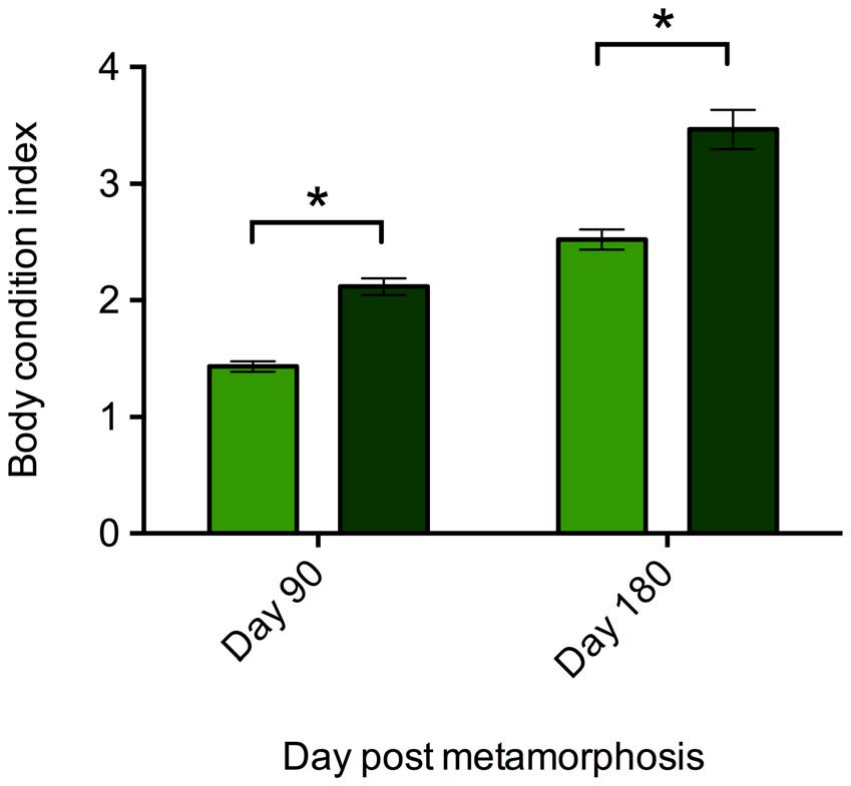
Body condition indices (BCI) of frogs housed under planted (dark green) and non-planted (light green) conditions. Error bars show ±1 S.E.M. An * indicates a significant (p<0.05) difference between treatment groups.

There was no significant effect of enclosures on SVL or BCI measures at any time point and this term was removed from GLMs. Frogs were housed in groups within enclosures, so a nested model was used to control for the effect of enclosure. There was no effect of enclosure, nested within treatment, on the SVL of frogs at the beginning (F _1,4_ = 2.425, p = 0.101), day 90 (F_1,4_ = 1.938, p = 0.164) or day 180 (F_1,4_ = 0.205, p = 0.931) of the experiment. Similarly, there was no significant effect on body condition on day 90 (F_1,4_ = 2.721, p = 0.076) or day 180 (F_1,4_ = 0.624, p = 0.654). We therefore ran proceeding models without the ‘enclosure’ term. There was no significant difference in SVL of frogs in planted and non-planted treatments at the start of the experiment (F_1,17_ = 1.617, p = 0.221). However, the SVL of frogs in planted tanks was significantly larger than that of frogs in non-planted tanks on day 90 (F_1,17_ = 22.039, p<0.001) and day 180 (F_1,17_ = 4.585, p = 0.047; Figure 3). At both days 90 and 180, frogs maintained in planted tanks showed significantly higher BCI values than frogs in non-planted tanks (Day 90: F_1,17_  = 69.836, p<0.001; Day 180: F_1,17_  =  26.636, p <0.001; Figure 4) indicating frogs maintained in planted enclosures had a significantly higher body condition.

### Bacterial communities

Overall, 29 bacterial morphotypes were isolated (Table 2), but one was not identified due to poor sequence data. Sequence data are available on the NCBI database (http://blast.ncbi.nlm.nih.gov/Blast.cgi) and accession numbers are listed in Table 2. Only one bacterial species was found in common between the two phases. The predominant families of bacteria across the two phases include *Comamonadaceae, Enterobactereacae*, and *Flavobacteriaceae*.

**Table 2:** Bacteria isolated from *Agalychnis callidryas* maintained in tanks with and without plants (*Sciandapsus sp*; Phase 1), and after a different plant species (*Dieffenbachia sp.*) had been introduced to all tanks (Phase 2).

Bacteria		Phase 1		Phase 2	
Family	Species (accession number)	Non-planted	Planted	Previously non-planted	Plant species changed
Acetobacteraceae	*Roseomonas sp. (KC853119)*	√	√		
Brevibacteriaceae	*Brevibacterium sp. (KC853124)*			√	√
Caulobacteraceae	*Brevundimonas sp. (KC853130)*			√	√
Comamonadaceae	*Acidovorax sp. (KC853129)*			√	√
Comamonadaceae	*Comamonas sp. (KC853135)*			√	√
Comamonadaceae	*Comamonas sp. (KC853116)*	√	√		
Comamonadaceae	*Comamonas sp. (KC853122)*	√	√		
Deinococcaceae	*Deinococcus sp. (KC853133)*			√	√
Dietziaceae	*Dietzia sp. (KF444797)*	√	√		
Enterobactereacae	*Citrobacter sp. (KC853113)*	√	√		
Enterobactereacae	*Enterobacter sp. (KC853127)*			√	√
Enterobactereacae	*Enterobacter sp. (KF444798)*			√	√
Flavobacteriaceae	*Chrysobacterium sp. (KC853131)*			√	√
Flavobacteriaceae	*Chryseobacterium sp. (KC853108)*	√	√		
Flavobacteriaceae	*Empedobacter sp. (KC853112)*	√	√		
Flavobacteriaceae	*Flavobacterium sp. (KC853132)*			√	√
Methylobacteriaceae	*Methylobacterium sp. (KF444799)*			√	√
Micrococcaceae	*Micrococcus sp. (KC853128)*			√	√
Moraxellaceae	*Acinetobacter sp. (KC853109/KC853125)*	√	√	√	√
Moraxellaceae	*Acinetobacter sp. (KC853110)*	√	√		
Paenibacillaceae	*Paenibacillus sp. (KC853136)*			√	
Sphingomonadaceae	*Sphinobacterium sp. (KC853121)*	√	√		
Staphylococcaceae	*Staphylococcus sp. (KC853111)*	√	√		
Staphylococcaceae	*Staphylococcus sp. (KC853118)*	√	√		
Tsukamurellaceae	*Tsukamurella sp. (KC853115)*	√	√		
Xanthomonadaceae	*Lysobacter sp. (KC853126)*			√	√
Xanthomonadaceae	*Stentrophomonas sp. (KC853134)*			√	√
	*Unidentified*	√	√		

#### Phase 1

**Figure 5:**
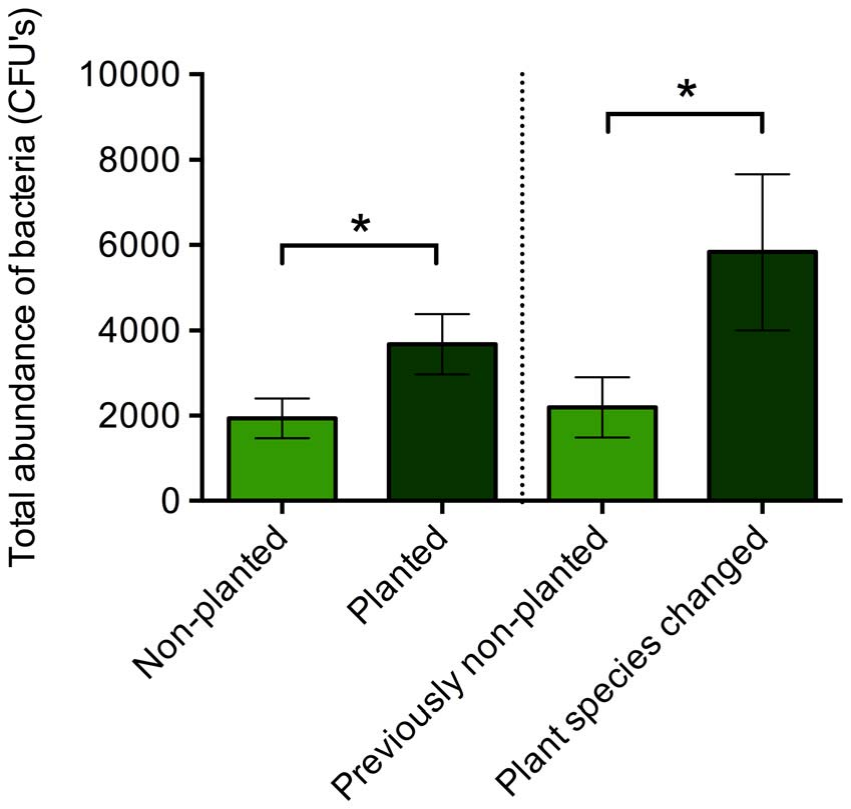
Total abundance of colony forming units (CFU's) of bacteria isolated from *Agalychnis callidryas* frogs maintained in non-planted and planted (with *Sciandapsus* plants) conditions (Phase 1; left hand side of dotted line), and after introduction of *Dieffenbachia* plants to all tanks (Phase 2; right hand side of dotted line). Error bars show ±1 S.E.M. An * indicates a significant (p<0.05) difference between treatment groups.

**Figure 6:**
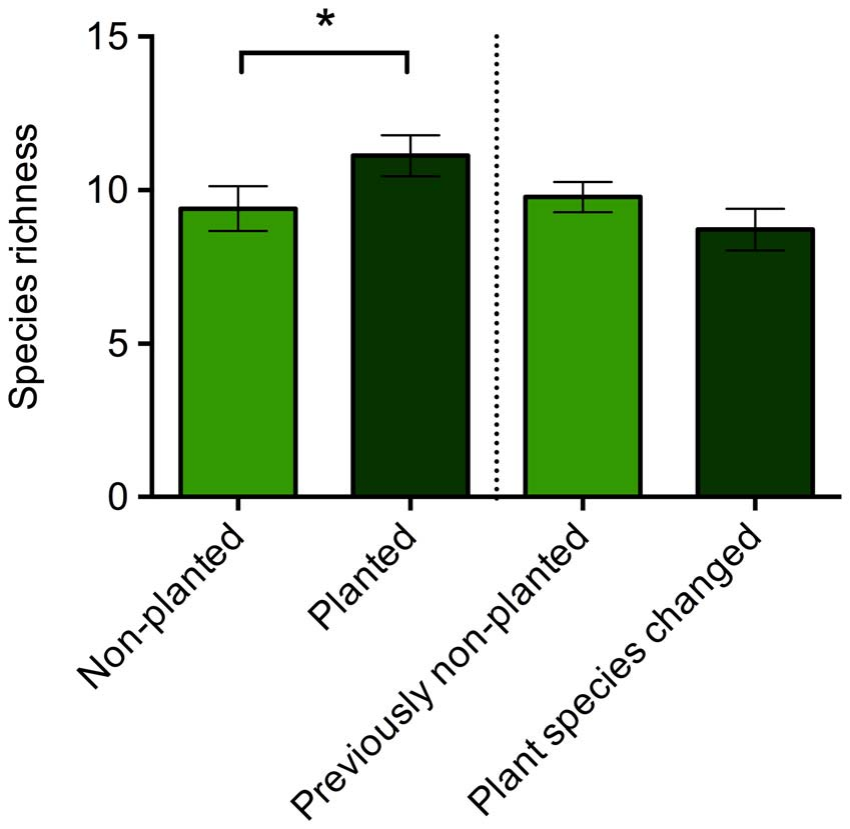
Species richness (number of different bacterial morphotypes) of bacterial community isolated from *Agalychnis callidryas* frogs maintained in non-planted and planted (with *Sciandapsus* plants) conditions (Phase 1; left hand side of dotted line), and after introduction of *Dieffenbachia* plants to all tanks (Phase 2; right hand side of dotted line). Error bars show ±1 S.E.M. An * indicates a significant (p<0.05) difference between treatment groups.

A total of 14 bacteria morphotypes were cultured, with a range of 7 to 13 morphotypes per individual. There were significant differences in the overall composition of the bacterial community associated with *A. callidryas* maintained with and without *Sciandapsus* plants (Adonis: F_1,16_  =  2.656, p  =  0.020). Frogs in the planted environment supported a significantly greater bacterial load (t_16_  =  2.213, p  =  0.025; Figure 5) and significantly greater species richness (t_16_  =  1.699, p  =  0.050; Figure 6). Group housing (‘tank’) did not have a statistically significant effect on species richness (F_5,12_  =  1.026, p  =  0.445), bacterial abundance (F_5,12_  =  2.607, p  =  0.091), or the Adonis analyses (F_1,17_  =  2.037, p  =  0.076).

#### Phase 2

A total of 15 bacteria morphotypes were cultured, with a range of 6 to 12 morphotypes per individual. After the addition of *Dieffenbachia* plants to all tanks there were no significant differences in the overall composition of the bacterial community associated with *A. callidryas* previously kept with and without *Sciandapsus sp*. (Adonis: F_1,14_  =  1.662, p  =  0.162). There was no significant difference in species richness (t_15_  =  1.298, p  =  0.892; Figure 6), although was a significant difference in the total abundance of bacteria (t_15_  =  2.032, p  =  0.031; Figure 5), with frogs previously maintained with *Sciandapsus* plants supporting a greater bacterial load. Group housing (‘tank’) did not have a statistically significant effect on species richness (F_5,11_  =  1.197, p  =  0.377), bacterial abundance (F_5,11_  =  1.069, p  =  0.432), or the Adonis analyses (F_1,14_  =  0.942, p  =  0.414).

Although there were no statistically significant differences in species richness between the two groups during Phase 2 of the study, the group previously kept with *Sciandapsus* plants and then changed to *Dieffenbachia* plants experienced a decrease in species richness (Figure 6). In order to determine whether this was related to the change in plant species, a small additional study was run using a third group of adult frogs (N  =  20), with half experiencing the same change in plant species (*Sciandapsus* to *Dieffenbachia*) and half maintained on *Sciandapsus* for the duration. These frogs were swabbed for bacterial communities (using exactly the same methods as before) both prior to the plant change and then again one month later. A two-way ANOVA showed there was no significant effect of sampling point (pre- or post-plant change), treatment group (plant species changed and plant species not changed) or their interaction on species richness (F_3,39_  =  0.157, p  =  0.925). However, both before and after the plant change, the average species richness for frogs where the plant species remained the same (7.5±0.5 and 7.8±0.2 respectively) and for those where the plant species was changed (7.3±0.7 and 7.4±0.3 respectively) was closer to the values obtained from the group of frogs that had the plant species changed (8.7±0.7) during Phase 2, compared to those that had plants introduced to their enclosure during Phase 2 (9.8±0.5). In addition, a two-way ANOVA model for sampling point, treatment group and their interaction was statistically significant (F_3,39_  =  6.461, p  =  0.001), with a Tukey's post-hoc analysis showing frogs in tanks that changed plant species had significantly increased bacterial abundance one month after the plant change. This is consistent with the results from the frogs in Group 1 in Phase 2 that also had their plant changed.

### Behavioural assay

Adults given a choice between planted and unplanted ends of an enclosure showed a significant preference for the planted end, with the mean position score for the population (0.872) being significantly greater than a score expected for random selection (0.5) (Wilcoxon Signed Rank, Z_29_  =  186.500, p < 0.001).

When adult frogs from a previous treatment of non-planted conditions in their home enclosure where given a choice between planted and unplanted ends of an enclosure (behavioural assay 2) they showed a significantly higher mean position score (0.989) than frogs with plants present in their home enclosure (0.756) (t_28_  =  3.394, p < 0.001). The variance was also significantly smaller for this group (F_14_  =  27.033; p<0.05) indicating these frogs show a more consistent response than those that came from planted home enclosures.

## Discussion

### Environmental parameters

Our results show that plant cover creates a more heterogeneous environment in enclosures. Plant leaves create shade from both UVB and infrared radiation, creating cool spots with low UV indices, even towards the top of the tank. They also reduce evaporation rates from the substrate, presumably by creating buffer zones of humid air protected from reduced air circulation, as well as by releasing water vapour through stomata by transpiration. Walsh and Downie [7] also found that shelters (coconut husk hides) provided to terrestrial frogs created more humid microclimates. Our measurements of frog skin temperature and resting position indicate that the provision of plants in enclosures allows frogs to better control their body temperature and exposure to UV light through behaviour. This may also have allowed frogs to regulate body temperature and other parameters while remaining towards the top of tanks, where they typically prefer to rest, rather than needing to descend to the floor to reduce body temperature or UVB exposure. Although variation in skin temperature was much larger in frogs maintained in planted tanks, median skin temperature was not significantly different between treatments, so temperature-dependent differences in metabolic rate may not have been important in causing differences in growth rates and body condition.

### Growth and body condition

Our data show that the provision of plant cover in the tank leads to significantly increased body size and BCI of *A. callidryas* relative to enclosures with no cover provided. At 6 months, frogs maintained under non-planted conditions were nearly 10% smaller in terms of mean SVL than frogs maintained under planted conditions, with nearly 20% lower body condition. This effect could be due to reduced foraging efficiency, as frogs without plants may have reduced hunting positions and so expend more energy per prey item, or due to frogs housed without plants investing more time in vigilance or anti-predator behaviours and less time foraging [7], [10]. Alternatively, stress from more homogenous and less hospitable environmental parameters may have reduced feeding rates [37], [38], [39], increased metabolic rate to fuel elevated immune function (which is often associated with increased stress; [37]) or a combination of both. Walsh and Downie [7] found significant effects of cover provision on the growth of three non-model study species (*Mannophryne trinitatis, Leptodactylus fuscus* and *Physalaemus pustulosus*). The authors attributed these differences to more favourable microclimates provided by the cover and to the effects of being able to hide from perceived predation risk [7]. In contrast to our data, tube or plant cover has minimal to no effect on growth rates of *X. laevis* [11], [12], [14] or body condition [14]. However, *X. laevis* is well known for its adaptability, which may allow it to cope more easily with a lack of cover.

The relatively large effect of plant cover on *A. callidryas* growth shown in the paper presented here is likely to represent an important real-world fitness cost, influencing survivorship both as adults and juveniles, as well as impacting on fecundity [26], [27], [31], [40]–[45]. In *A. callidryas*, body size is linked to fitness traits in both males (quality of offspring; [26]) and females (egg counts, egg size variation and egg volume; [27]). Faster growing juveniles will reach a larger adult body size than slower growing conspecifics [41], [42] and are therefore likely to derive a reproductive and survival advantage [43], which may be relevant to conservation programs involving the release of juvenile animals into the wild. In addition, lower body condition, as well as smaller body size, is associated with reduced survivorship [40], [44], reduced fecundity [40] and reduced reproductive output [45].

### Bacterial communities

The presence of *Sciandapsus* plants had a significant effect on the bacterial community associated with frogs in terms of overall community composition, species richness, and total abundance of bacteria. *Agalychnis callidryas* maintained with *Sciandapsus* plants during Phase 1 of the study had a significantly greater diversity (abundance and richness) of bacteria associated with their skin than *A. callidryas* maintained without plants. A richer bacterial community is likely to be advantageous as this may increase the stability and productivity of the community, making it less susceptible to pathogenic infection, and it may also increase the potential for the presence of a species that can protect the host from pathogens such as the chytrid fungus [18], [46]–[48]. Moreover, chemical signalling between bacteria (quorum sensing) means that a high abundance of bacteria may be important for initiating antibiotic defences and/or producing the minimum inhibitory concentrations required to protect the host from invasive pathogens [19], [49], [50].

The differences in the bacterial communities may be linked to intrinsic factors, such as differences in peptide secretion as a result of alter perceived vulnerability or stress [51]–[53], or extrinsic factors, such as the bacterial community associated with the plants, or the differences in abiotic conditions between the two the environments as described above. Loudon et al. [54] found that red-backed salamanders (*Plethodon cinereus*) maintained with either soil collected from the wild or sterile Provasoli medium supported different bacterial communities after 28 days, indicating the environment and its associated bacteria (or lack of) affects the community associated with the host amphibian. However, given that the same species of bacterial morphotypes were isolated from frogs maintained in both planted and non-planted environments (see Table 2), it is unlikely that differences in the bacterial communities were due to differences in the environment (i.e. the presence or lack of plants), and differences are more likely linked to variation in abiotic conditions, or some physiological response from the frogs to the different environments.

It is worth noting, as bacterial samples were not taken at the start of experiment, frogs in treatment groups may have had different bacterial communities initially. However, as all frogs originated from the same clutch, were reared as one group, were randomly assigned to treatment group, and all conditions were exactly the same between the two treatment groups with the exception of the presence or absence of plants, we believe the effects observed can only be attributed to differences in the experimental manipulations.

The results from Phase 2 of the bacterial study show that when *Dieffenbachia* plants are introduced to tanks containing *A. callidryas* previously maintained with and without *Sciandapsus*, the overall bacterial community and the bacterial species richness associated with the two groups of frogs are no longer significantly different. In the case of species richness, it is worth noting that this stayed relatively similar for the group that was previously maintained without plants (see Figure 6), whereas the species richness decreased for the group that had the plant species changed. Moreover, there is a significantly greater total abundance of bacteria associated with *A. callidryas* maintained with plants throughout their lives than those previously kept without plants. The results of the plant change study with the third group of frogs indicate that a change in plant species does not cause a significant change in bacterial species richness, and the bacterial morphotypes are not different between the groups. In addition, the average species richness from all treatment groups of this third group of adult frogs is more similar to the species richness obtained from the adult frogs during Phase 2 of the original plant change study, rather than the species richness obtained from these frogs during Phase 1 when they were juveniles. This suggests the fall in species richness observed in Group 1 frogs that had their plant species changed was more likely caused by maturation of the frogs (or some other unidentified cause) than the plant change itself, which may have been buffered in frogs previously without plants when these were introduced in Phase 2. However, changing the plant species does cause a subsequent increase in the bacterial abundance for frogs, which lasts for months after the event. Whether this is of benefit or detriment to frogs remains to be tested.

Only one bacterial morphotype was found in common between Phases 1 and 2 of the study (*Acinectobacter sp.*), although there were some similarities between the two phases at the family and genus level (e.g. *Comamonas, Acinetobacter, Chryseobacterium*; see Table 2). Changes in the individual bacterial species isolated from each phase of this study are unlikely to be due to the change in the plant species because in the third group of frogs, the same morphotypes were collected from frogs that did and did not have their plant species changed. Changes in the bacterial community over time are more likely a result of variation throughout development and maturation. Culturing methods are known to greatly underestimate species richness and bacterial abundance (reviewed in [55]) and therefore the responses of the remainder of the bacterial community to the presence or absence of plants is unknown. However, this study provides insight into the effects of a planted environment on a subset of the bacterial community present of the skin of amphibians, which may be applicable to the rest of the non-culturable community. Overall these results are likely to have implications for the ability of amphibians to retain probiotic treatments (which are currently identified using culturing techniques) and more work is required to determine how the bacterial community associated with amphibians alters throughout maturity.

### Behavioural data

We found that *Agalychnis callidryas* adults show a significant preference for planted over non-planted conditions when offered a choice between these two conditions. Similar results have been found with other amphibian species, including *X. laevis*, where preference was exhibited for sheltered environments, particularly plastic tubes, with a subsequent reduction in clumping behaviour, daytime activity, aggressive encounters and subsequent injury [11]–[15], [56]. Walsh and Downie [7] also found the three non-model study species of frog (*M. trinitatis, L. fuscus* and *P. pustulosus*) spent more time under shelters when available than in the open.

In our study, frogs that had been previously housed without plants clearly showed a stronger preference for planted enclosures than those previously housed with plants. This coupled with the overall preference for cover suggests that frogs seek out plants for shelter if given the option, and exhibit a stronger behavioural response once access to plants is restored after a period of plant absence. This is potentially an effort to alleviate some level of physiological stress experienced from lack of cover, although analysis of corticosteroid ‘stress’ hormones (see [57], [58]) would be required to confirm this hypothesis.

## Conclusions

In this study we found a large and positive effect of providing shelter to frogs on growth, body condition, bacterial communities and behaviour in captive amphibians, which are at least partly linked to an increased heterogeneity as a result of plants in enclosures. This suggests that captive enclosure design may influence the long-term fitness of captive populations, and the chances of success for reintroduction attempts. This is important for institutions of all types that maintain amphibians in captivity for both conservation and research. Our data also extends work on non-model amphibians to include arboreal anurans, which have thus far not been investigated, and calls to further attention the problems associated with using laboratory *X. laevis* as a model for designing enclosures for other amphibians. We used a single sibship of frogs in this study so, given the prevalence of clutch effects on a variety of characters in amphibians [59]–[61], there may be a greater variation in responses to cover treatments across multiple clutches. However, given the very strong responses to treatments across the range of measures used in this study, and the clear effects of plant cover on the physical environment that we detected, it is likely that planted cover should be important for any sibship of this species. We also suggest that this evidence can be applied to other tree frog species aside from *A. callidryas*, with a similar ecology, until more species-specific data is available. It is also important to expand this sort of research to include other non-model amphibians, particularly for those taxa and ecotypes that are not yet represented, including salamanders and caecilians.
